# Letter to the editor of Heliyon re: Grassmann, G. “New considerations on the validity of the Wiener-Granger causality test” [Heliyon 6 (2020) e05208]

**DOI:** 10.1016/j.heliyon.2021.e07948

**Published:** 2021-09-10

**Authors:** Martina Chvosteková, Anna Krakovská

**Affiliations:** Department of Theoretical Methods, Institute of Measurement Science of the Slovak Academy of Sciences, Dúbravská cesta 9, 841 04 Bratislava, Slovakia; Department of Complex Systems, Institute of Computer Science of the Czech Academy of Sciences, Pod Vodárenskou věží 2, 182 07 Prague 8, Czech Republic; Department of Theoretical Methods, Institute of Measurement Science of the Slovak Academy of Sciences, Dúbravská cesta 9, 841 04 Bratislava, Slovakia

Dear Editor,

a recently published article [1] questions the Wiener-Granger causality test, more commonly referred to as the Granger causality test. Although the abstract of the paper states that the results were obtained by mathematical tools such as the Fourier transform and differential calculus, the presented serious claim that “*...not even the most basic requirement underlying any possible definition of causality is met by the Granger causality test...*” is in fact based on a questionable numerical testing of two filtered neural signals (A and B). We will show that the validity of the presented considerations is unfounded for several reasons.

It is said that a time series $x=:{\left\{x_{t}\right\}}_{t=1}^{T}$ Granger causes a time series $y=:{\left\{y_{t}\right\}}_{t=1}^{T}$ if the variance (or, equivalently the standard deviation) of the prediction error of *y* including only past values of *y*

(1) $$\epsilon_{t}(y/y)=y_{t}-\sum_{j=1}^{p}\alpha_{j}y_{t-j}$$

is greater than the variance of the prediction error of *y* including jointly the past values of both *y* and *x*

(2) $$\epsilon_{t}(y/y,x)=y_{t}-\sum_{j=1}^{p}\beta_{j}y_{t-j}-\sum_{j=1}^{q}\gamma_{j}x_{t-j},$$

i.e., *x* is helpful in predicting *y*. *T* denotes the length of the time series. Granger showed that a time series *y* is not Granger-caused by a time series *x* if and only if all coefficients at past values of *x* in the linear joined prediction model of $y_{t}$ (i.e., $\gamma_{j}$'s in Equation (2)) are zero [2]. Other equivalent conditions and corresponding alternative testing options are discussed in [3]. Most often, however, Granger's causality is statistically evaluated by the *F*-test of significance of regression coefficients [4]. If the correlation structure of *x* and *y* is adequately captured by an autoregressive model of a finite reasonable order, then the *F*-test is an exact (or robust) test, i.e. the probability of a false detection by the *F*-test is equal to a chosen significance level *α*. The power of the *F*-test, defined as a complement to the probability of a false negative detection, depends on the length of the time series. The power increases to 1 by increasing *T*.

In [1], instead of the *F*-test (or any statistical test), the causal link between time series is inferred after just comparing the standard deviation estimates of the predictive errors, i.e. $\sigma (\epsilon_{t}(y/y))$ and $\sigma (\epsilon_{t}(y/y,x))$ to each other. Such an approach does not provide a statistical significance of a prediction improvement. Statistical inference is important in order to provide a conclusion about the underlying population parameters based on a sample of the data. Another problem is that the root mean square (RMS) given as

(3) $$RMS=\sqrt{\frac{\sum_{t=p^{\prime }+1}^{T}\epsilon_{t}^{2}}{T^{\prime }}},\;\;p^{\prime }=max(p,q),\;T^{\prime }=T-p^{\prime },$$

as an used estimator of standard deviation in [1], spuriously decreases by adding a variable to a prediction as can also be seen, e.g., in Figures 6, 7 of the paper [1]. Then, it is clear that to compare the estimated $\sigma (\epsilon_{t}(y/y))$, $\sigma (\epsilon_{t}(y/y,x))$ by RMS is meaningless and such test is not suitable for analysing the Granger causality at all. Note that in a regression with nonstochastic regressors, RMS is a biased estimator of standard deviation, see e.g. [5]. An unbiased estimator can be obtained decreasing the number of the model's data $T^{\prime }$ by the number of coefficients in a linear prediction, i.e. by *p* for $\epsilon_{t}(y/y)$, and by p+q for $\epsilon_{t}(y/y,x)$. If the unbiased estimator, here referred as the root adjusted mean square (RAMS), was used, it may be possible to reach a different conclusion about the causal connection between the observed time series.

Let us explain all of this on a simple example of unidirectionally (x→y) linearly coupled time series defined as

(4) $$x_{t}=\alpha_{1}x_{t-1}+\alpha_{2}x_{t-2}+\epsilon_{t}(x)y_{t}=\beta_{1}y_{t-1}+\beta_{2}y_{t-2}+0.1x_{t-2}+\epsilon_{t}(y),$$

where coefficients $\alpha_{1},\alpha_{2},\beta_{1},\beta_{2}∼U(-1,1)$ are uniformly generated on interval [−1,1], $\epsilon_{t}(x),\epsilon_{t}(y)∼N(0,0.5^{2})$ are identically normally distributed zero-mean variables with common (time invariant) variance equal to 0.5^2^. Since the errors $\epsilon_{t}(x),\epsilon_{t}(y)$ represent zero mean white noise vector processes (serially independent) and coefficients were chosen so that both time series were stable, the processes (4) satisfy the assumptions for Granger causality analysis. We analyzed the performance of the *F*-test at significance level α=5% and two non-statistically made decisions (one with RMS and one with RAMS) on 10000 simulations of the time series (4). Fig. 1 shows the number of detections of a certain link between time series *x*, *y* by the three studied approaches.

**Figure 1:**
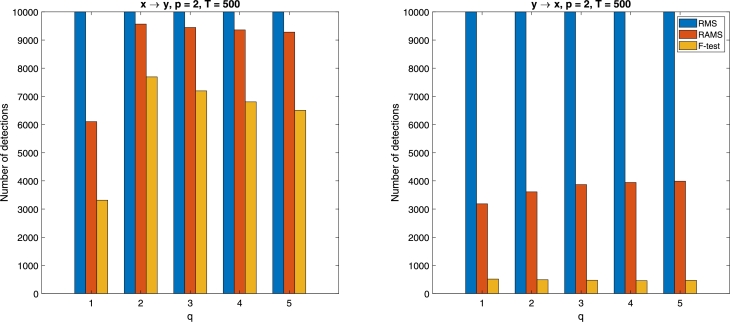
Number of correct detections of the causal link *x* → *y* (on the left) and false detections of *y* → *x* (on the right) obtained by the made decision with RMS used in [1], by the made decision with RAMS, and by the *F*-test (*α* = 5 %) from 10000 simulations of the time series (4), *p* = 2, *T* = 500, *q* = {1,2,…,5}.

We can see in Fig. 1 that the probability of a false positive detection of the non-statistical decision with RMS equals to one. In contrast to the RMS results, the test with RAMS incorrectly detected the causal link from *y* to *x* in approximately 40% of the simulations and failed to detect the true causal link from *x* to *y* in approximately 5% of simulations. The false positive rate for the *F*-test equals to the chosen significance level α=5% independently of the value of *q* and its power (ability to detect true causal link) is around 70% for our simulations. Fig. 1 demonstrates the fact that RMS falsely decreases even by adding the past value of causally independent time series to the linear joined prediction, and the non-statistical decision with RMS is therefore completely unsuitable for inferring Granger causality.

The second problem is that at least the filtered signal B violates the fundamental assumption of the Granger causality definition. The Granger causality test involved only stochastic stationary processes which can be fitted by a linear autoregressive model. A stationarity is required since a causal relation is analyzed by comparing the variance of predictive error of the linear predictions. In the non-stationary case, the variances will depend on time and the existence of causality may alter over time. Assuming that a stable process has been initialized in the infinite past, the process is stationary and ergodic with time invariant mean, variance, and autocovariances [6]. So, one way to determine if an autoregressive model of order *p* (AR(p)) is stationary is to check if the roots of its characteristic equation lie outside the unit circle. We analyzed the stability of non-filtered signal B and two filtered versions of signal B fitted by AR(4). Table 1 shows the obtained coefficients (i.e., the values from Table 3 in [1]) and the computed roots of their characteristic equations. It follows from Table 1 that the non-filtered B signal seems to be a stable process (i.e., all four roots lie outside the unit circle), while the fitted filtered signal B with any of two cutting frequencies is unstable (i.e., at least one of the roots lies inside or on the border of the unit circle). The filtration led to the violation of stability assumption for the signal B. Moreover, the signal B filtered with the cutting frequency 0.008 led to a non-stochastic (deterministic) function. In general, if a value of time series is perfectly predictable from its own past, clearly no value from another time series can improve the prediction [3]. Consequently, the Granger causality test is no valid with any of two filtered signals B.

**Table 1:** Coefficients of AR(4) from Table 3 in [1] and the roots of the characteristic equations of the fitted AR(4).

Non-filtered signal B	
AR coefficients	Roots

0.9393, 0.0077,	3.5321 + 0i, -1.1915 + 2.3763i
0.0858, -0.0398	-1.1915 - 2.3763i, 1.0066 + 0i

Signal B with cutting frequency at 0.16	

AR coefficients	Roots

2.9904, -3.7965	0.66829 + 0.87492i, 0.66829 - 0.87492i
2.5387, -0.7342	1.095 + 0i, 1.0262 + 0i

Signal B with cutting frequency at 0.008	

AR coefficients	Roots

3.8981, -5.7480	0.9836 + 0.22857i, 0.9836 - 0.22857i
3.8001, -0.9502	1.0321 + 0i, 1 + 0i

Another problem is how the concept of testing the time inverted signal A is discussed. According to the results of RMS analysis it was stated in [1] that the future of the signal B is equally well predicted from the past of A as the past of B is predicted from the future of A, where the time inverted signal A represented the “future” of the signal A. However, it is not an evidence that the past of B can be equally well predicted from the future of A if the values from the time inverted signal A are included to the linear joined prediction of B. Indeed, the filtered signal B was not actually predicted from a future of A by using the time inverted signal A. Let us show this on an example of two short time series $a:=\{a_{1},a_{2},a_{3},a_{4},a_{5}\}$ and $b:=\{b_{1},b_{2},b_{3},b_{4},b_{5}\}$. Consider that p=q=2 and that *b* is predicted from two own past values and with two past values from the time inverted *a*, i.e., $\left\{a_{5},a_{4},a_{3},a_{2},a_{1}\right\}$. It implies that the value $b_{3}$ is predicted using a linear combination of $b_{1},b_{2},a_{5},a_{4}$, the value $b_{4}$ is predicted using a linear combination of $b_{2},b_{3},a_{4},a_{3}$, and the value $b_{5}$ is predicted using a linear combination of $b_{3},b_{4},a_{3},a_{2}$ (values $b_{1}$, $b_{2}$ cannot be obviously predicted according to the chosen orders of the prediction model). Which means that value $b_{3}$ is predicted from the future of *a*, while values $b_{4}$, $b_{5}$ are not.

This brings us to the last problem of misinterpretation of the role of the Axiom A [3]: *“The past and present may cause the future, but the future cannot cause the past”*. The author in [1] argued that the Granger causality test is in contradiction with the Axiom A since it was found that the future of signal A (i.e., the time inverted signal A in meaning of the author) is helpful for the prediction of the past B. We have already shown that the validity of this argument is questionable due to the previous mentioned reasons, but the implication is invalid in principle. It must be emphasized that Axiom A was this axiom that allowed Granger to build a testable concept of causality, where stochastic nature of time series and the direction of the flow of time play a central role. The origin of the theory can be found in work of Wiener [7]. According to the Wiener-Granger theory the causal connection from *x* to *y* is evaluated by analysing of the prediction improvement and a prediction can be based only on the past values of *y* or the past of both two time series. The Granger causality test is done for a data under acceptance the Axiom A and therefore the truth of Axiom A for the data cannot be tested using a Granger causality test, see [3]. It is possible to discuss whether, after the time reversal of both two unidirectionally connected time series and the subsequent causal analysis, the detected causality also reverses [8]. However, Granger's definition of causality says nothing about improving the prediction of *y* from the future values of *x*. There is no reason to expect, as the author did in [1], that the causal link from *x* to *y* will disappear after using the future of *x* instead of its past in the linear joined prediction of *y* in general (it may depend on the prediction precision of *x* and *y* from their own past, on the strength of causal connection in the original order, and also on how far future values of *x* are used in the linear joined prediction of *y*).

We believe that the paper [1] did not change the fact that the Granger causality test is a useful tool, if applied correctly, on appropriate data and with subsequent careful interpretation.

## Declarations

### Author contribution statement

All authors listed have significantly contributed to the development and the writing of this article.

### Funding statement

Anna Krakovská was supported by the 10.13039/501100006109Scientific Grant Agency of the Ministry of Education, Science, Research and Sport of the Slovak Republic and Slovak Academy of Sciences (VEGA 2/0081/19); Martina Chvosteková was supported by the 10.13039/501100006109Scientific Grant Agency of the Ministry of Education, Science, Research and Sport of the Slovak Republic and Slovak Academy of Sciences (VEGA 2/0081/19, VEGA 2/0096/21) and the 10.13039/501100004240Czech Academy of Sciences (Praemium Academiae awarded to M. Paluš).

### Data availability statement

Data included in article/supplementary material/referenced in article.

### Declaration of interests statement

The authors declare no conflict of interest.

### Additional information

No additional information is available for this paper.
